# Predictors of *de novo* atrial fibrillation in a non-cardiac intensive care unit

**DOI:** 10.5935/0103-507X.20180022

**Published:** 2018

**Authors:** João Bicho Augusto, Ana Fernandes, Paulo Telles de Freitas, Victor Gil, Carlos Morais

**Affiliations:** 1 Serviço de Cardiologia, Hospital Professor Doutor Fernando Fonseca - Lisboa, Portugal.; 2 Unidade de Cuidados Intensivos Polivalente, Hospital Professor Doutor Fernando Fonseca - Lisboa, Portugal.; 3 Unidade Cardiovascular, Hospital dos Lusíadas - Lisboa, Portugal.

**Keywords:** Atrial fibrillation/epidemiology, Incidence, Intensive care

## Abstract

**Objective:**

To assess the predictors of *de novo* atrial fibrillation in patients in a non-cardiac intensive care unit.

**Methods:**

A total of 418 hospitalized patients were analyzed between January and September 2016 in a non-cardiac intensive care unit. Clinical characteristics, interventions, and biochemical markers were recorded during hospitalization. In-hospital mortality and length of hospital stay in the intensive care unit were also evaluated.

**Results:**

A total of 310 patients were included. The mean age of the patients was 61.0 ± 18.3 years, 49.4% were male, and 23.5% presented *de novo* atrial fibrillation. The multivariate model identified previous stroke (OR = 10.09; p = 0.016) and elevated levels of pro-B type natriuretic peptide (proBNP, OR = 1.28 for each 1,000pg/mL increment; p = 0.004) as independent predictors of *de novo* atrial fibrillation. Analysis of the proBNP receiver operating characteristic curve for prediction of *de novo* atrial fibrillation revealed an area under the curve of 0.816 (p < 0.001), with a sensitivity of 65.2% and a specificity of 82% for proBNP > 5,666pg/mL. There were no differences in mortality (p = 0.370), but the lengths of hospital stay (p = 0.002) and stay in the intensive care unit (p = 0.031) were higher in patients with *de novo* atrial fibrillation.

**Conclusions:**

A history of previous stroke and elevated proBNP during hospitalization were independent predictors of *de novo* atrial fibrillation in the polyvalent intensive care unit. The proBNP is a useful and easy- and quick-access tool in the stratification of atrial fibrillation risk.

## INTRODUCTION

The prevalence of atrial fibrillation (AF) is high, reaching 10% in individuals over 80 years of age.^(^^1^^-^^3^^)^ AF is associated with longer stays in the hospital and intensive care unit (ICU),^(^^2^^,^^4^^)^ and *de novo* AF in critically ill patients is associated with higher mortality.^(^^5^^)^ The clinical complexity of patients in the ICU requires rapid diagnosis and effective treatment of this condition.^(^^6^^-^^8^^)^

In this context, knowledge of the epidemiology of this event in critically patients becomes important. The incidence of *de novo* AF ranges from 5 to 65%, depending on the type of ICU, and is higher in patients undergoing cardiac surgery.^(^^9^^-^^18^^)^ In turn, the large variation in the incidence of *de novo* AF in the various types of ICU can be explained by different predictors of AF occurrence.

Some of these predictors of *de novo* AF have already been described in the literature, especially in critical cardiac patients, such as advanced age, greater severity score on admission, surgical or post-trauma admission, occurrence of sepsis, and need for ventilatory or catecholamine support. However, for medical and non-cardiac surgical ICU patients, there is a paucity of data in the literature regarding predictors of *de novo* AF.^(^^4^^)^

Therefore, the objective of this study was to investigate the predictive factors of *de novo* AF in patients in a non-cardiac polyvalent ICU (critically ill and non-cardiac surgical patients). As secondary objectives, the incidence of *de novo* AF and its prognostic impact in terms of in-hospital mortality and length of hospital and ICU stay were also evaluated.

## METHODS

A sample of patients hospitalized during a period of 9 months (January 1, 2016 to September 30, 2016) in a non-cardiac polyvalent ICU at the Fernando Fonseca Hospital, Lisbon, Portugal, were retrospectively and consecutively analyzed.

The data were obtained through clinical consultations and were complemented by analytical and other diagnostic evaluations. The Hospital Ethics Committee approved the study, and informed consent was not required given the study's observational nature.

The ICU had 14 beds. Patients with a pathology requiring mechanical ventilation, trauma patients, and non-cardiac surgery patients were admitted.

All patients were under continuous cardiac monitoring with three leads. The presence of an absolutely irregular RR interval with no apparent P waves or the replacement of these by AF waves was classified as AF, with subsequent confirmation on a 12-lead electrocardiogram. For classification as *de novo* AF, all patients with sinus rhythm on ICU admission and without any record of prior AF or atrial flutter (documented electrocardiographically, in a previous medical report or indicated by the patient and/or family) were considered. To this end, the national platform of medical records, called the Health Data Platform (Plataforma de Dados da Saúde), was also consulted. Patients with a definite pacemaker on admission or previous cardiac surgery, chest trauma, or pulmonary thromboembolism in the last year (the latter two associated with a higher risk of *de novo* AF) were excluded from this group.

Each patient was classified according to the reason for hospitalization: medical, surgical, or trauma. Each patient was further stratified on admission according to the in-hospital mortality scores Acute Physiology and Chronic Health Evaluation II (APACHE II)^(^^19^^)^ and Simplified Acute Physiology Score (SAPS II).^(^^20^^)^

The presence of the following cardiovascular disease and risk factors was evaluated: arterial hypertension, dyslipidemia, diabetes mellitus, obesity, smoking, heart valve disease, heart failure (HF), and previous acute coronary syndrome. Individuals with at least 1 year of smoking cessation were considered former smokers/non-smokers. The presence of heart valve disease was assumed in individuals with stenosis and/or moderate or severe failure of at least one valve, previously documented by an imaging method. Chronic obstructive pulmonary disease (COPD), obstructive sleep apnea syndrome, stroke, thyroid function disorders, and chronic kidney disease were also documented. The COPD definition of the Global Initiative for Chronic Obstructive Lung Disease was adopted.^(^^21^^)^ In cases of kidney injury or a glomerular filtration rate of less than 60mL/min/1.73m^2^ for 3 months or more, the presence of chronic kidney disease was assumed, according to the Kidney Disease Outcomes Quality Initiative of the National Kidney Foundation.^(^^22^^)^ The infectious complications were recorded (nosocomial infection, sepsis, and septic shock), applying the criteria defined by the recommendations of the Surviving Sepsis Campaign.^(^^23^^)^

Information regarding the interventions performed up to the date of occurrence of AF were recorded. The peak/maximum values of C-reactive protein, serum creatinine, and pro-B type natriuretic peptide (proBNP) were also documented in all patients admitted to the ICU, as was the serum albumin nadir/minimum value during hospitalization until the date of occurrence of AF. Serial measurements of these biomarkers are part of the institutional protocol.

### Statistical analysis

The demographic and clinical characteristics of the sample were analyzed using descriptive statistics. Continuous variables with normal distribution are expressed as the mean ± standard deviation (SD), and categorical variables as the number of patients in each category and corresponding percentages. Nonparametric continuous variables are expressed as medians and interquartile ranges. Normal distribution was assessed using the Kolmogorov-Smirnov test.

Continuous variables were compared using the independent Student's *t* test or Mann-Whitney U test, as appropriate. The association of categorical variables was assessed using the chi-square test or Fisher's exact test.

Univariate logistic regression analysis was used to identify risk factors associated with the development of *de novo* AF during ICU stay. All variables considered as significant predictors of *de novo* AF (p < 0.05) were further analyzed using multivariate logistic regression. The results of the regression analysis are expressed as odds ratios (ORs) and 95% confidence intervals (95%CI), with p < 0.05 being considered statistically significant.

The peak proBNP performance for prediction of *de novo* AF during ICU stay was tested using the receiver operating characteristic (ROC) curve. The Youden index was used to identify the optimal cutoff point of proBNP, thereby determining the sensitivity, specificity, accuracy, predictive values, and positive and negative likelihood ratios.

Lastly, the impacts of *de novo* AF on the lengths of stay in the hospital and ICU were analyzed using the Mann-Whitney U test, and its impact on in-hospital mortality was analyzed using the Fisher exact test.

Statistical analysis was performed with the Statistical Package for Social Science (SPSS), version 22.0 (Chicago, IL, USA).

## RESULTS

**Figure 1:**
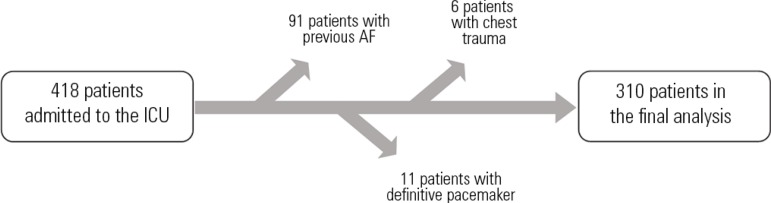
Flowchart of patient inclusion in the study. AF - atrial fibrillation; ICU - intensive care unit.

Of the 418 patients admitted to the ICU during the study period, 91 patients were excluded due to previous AF (21.8%), 11 due to the presence of a definitive pacemaker (3.4%), and 6 due to chest trauma (1.9%). No patient had a history of pulmonary thromboembolism in the last year or had been admitted to the ICU for cardiac surgery (Figure 1). Thus, 310 patients admitted during the study period were included in the final analysis.

The mean age of the patients was 61.0 ± 18.3 years, and 49.4% (n = 153) were male. Table 1 summarizes the main demographic and clinical characteristics of our sample.

**Table 1:** General population characteristics (n = 310)

Variables	
Age (years)	61.0 ± 18.3
Male sex	153 (49.4)
Type of admission	
Medical	242 (78.1)
Surgical	57 (18.4)
Non-thoracic trauma	11 (3.5)
Risk factor/cardiovascular pathology	
Arterial hypertension	166 (53.5)
Dyslipidemia	43 (13.9)
Diabetes mellitus	64 (20.6)
Obesity	23 (7.4)
Smoking	41(13.2)
Heart failure	35 (11.3)
Valve disease	7 (2.3)
Acute coronary syndrome	24 (7.7)
Respiratory disease	
Chronic obstructive pulmonary disease	41 (13.2)
Obstructive sleep apnea syndrome	8 (2.6)
Stroke	57 (18.4)
Chronic kidney disease	36 (11.6)
Thyroid dysfunction	10 (3.2)
APACHE II	16 (10 - 26)
SAPS II	36 (23 - 56)

SD - standard deviation; APACHE II - Acute Physiology and Chronic Health Evaluation II; SAPS II - Simplified Acute Physiology Score. Values are expressed as the means ± standard deviations, n (%), or medians (interquartile ranges).

Table 2 summarizes the outcomes, complications (nosocomial infection, sepsis, septic shock, and death), the interventions performed during hospitalization, and the values of the laboratory markers studied.

**Table 2:** Outcomes, complications, interventions performed, and laboratory markers (n = 310)

Infectious complications	
Nosocomial infection	129 (41.6)
Sepsis	152 (49.0)
Septic shock	71 (22.9)
Interventions	
Catecholamine support	90 (29.0)
Non-invasive ventilation	43 (13.9)
Invasive mechanical ventilation	168 (54.2)
Days on invasive ventilation	1 (0 - 7)
Reintubation	13 (4.2)
Tracheotomy	23 (7.4)
Renal replacement	31 (10.0)
Central venous catheter	221 (71.3)
Laboratory markers	
Peak serum creatinine (mg/dL)	1.37 (0.93 - 2.75)
Nadir serum albumin (g/dL)	2.85 ± 1.79
Peak C-reactive protein (mg/dL)	16.9 (6.3 - 28.8)
Peak proBNP (pg/mL)	4,640 (1,220 - 10,155)
Days of ICU stay	6 (3 - 13)
Days of hospital stay	9 (4 - 20)
In-hospital mortality	52 (16.8)

proBNP - pro-B type natriuretic peptide; ICU - intensive care unit. Values are expressed as n (%), medians (interquartile ranges), or means ± standard deviations.

During the study period, 73 patients with *de novo* AF (23.5%; 95%CI 18.9 - 28.7) were recorded. The incidence rates of *de novo* AF were 24.2% in males and 22.9% in females (p = 0.894). *De novo* AF occurred in 15.3% of medical admissions, 15.8% of surgical admissions, and 9.1% of admissions due to non-thoracic trauma.

Table 3 summarizes the general characteristics of the population, according to the presence or not of *de novo* AF (univariate analysis). Upon admission, patients with *de novo* AF were significantly older (70.1 ± 14.7 years *versus* 58.1 ± 18.5 years; p < 0.001) and had higher baseline prevalence rates of arterial hypertension (68.5% *versus* 48.9%; p = 0.005), HF (26.0% *versus* 6.8%; p < 0.001), valve disease (8.2% *versus* 0.4%; p = 0.001), stroke (27.4% *versus* 15.6%; p = 0.037), and thyroid dysfunction (8.2% *versus* 1.7%; p = 0.007). All 6 patients with *de novo* AF and thyroid dysfunction had hypothyroidism. The median APACHE II scores (21 points *versus* 15 points) and SAPS II scores (47 points *versus* 34 points) were also significantly higher in patients with *de novo* AF (p = 0.004 and p < 0.001, respectively).

**Table 3:** Sample characteristics according to the presence or absence of *de novo* atrial fibrillation

	*De novo* AF (n = 73)	No *de novo* AF (n = 237)	p value
Age in years	70.1 ± 14.7	58.1 ± 18.5	< 0.001
Male sex	37 (50.7)	116 (48.9)	0.894
Type of admission			
Medical	63 (86.3)	179 (75.5)	0.290
Surgical	9 (12.3)	48 (20.3)	
Non-thoracic trauma	1 (1.4)	10 (4.2)	
Risk factors/cardiovascular pathology			
Arterial hypertension	50 (68.5)	116 (48.9)	0.005
Dyslipidemia	12 (16.4)	31 (13.1)	0.446
Diabetes mellitus	12 (16.4)	52 (21.9)	0.408
Obesity	4 (5.5)	19 (8.0)	0.613
Smoking	8 (11.0)	33 (13.9)	0.693
Heart failure	19 (26.0)	16 (6.8)	< 0.001
Valve disease	6 (8.2)	1 (0.4)	0.001
Acute coronary syndrome	2 (2.7)	4 (1.7)	0.629
Respiratory disease			
Chronic obstructive pulmonary disease	9 (12.3)	32 (13.5)	1.000
Obstructive sleep apnea syndrome	3 (4.1)	5 (2.1)	0.398
Stroke	20 (27.4)	37 (15.6)	0.037
Chronic kidney disease	10 (13.7)	26 (11.0)	0.534
Thyroid dysfunction	6 (8.2)	4 (1.7)	0.007
APACHE II	21 (12 - 28)	15 (10 - 24)	0.004
SAPS II	47 (33 - 65)	34 (22 - 51)	< 0.001

AF - atrial fibrillation; APACHE II - Acute Physiology and Chronic Health Evaluation II; SAPS II - Simplified Acute Physiology Score. Values are expressed as the means ± standard deviations, n (%), or medians (interquartile ranges).

Table 4 summarizes the complications, interventions performed during hospitalization, and values of the laboratory markers studied, according to the presence or not of *de novo* AF (univariate analysis). Patients with *de novo* AF had a higher prevalence of septic shock (37% *versus* 18.6%; p = 0.007) and a greater need for catecholamine support (41.1% *versus* 25.3%; p = 0.012) and central venous catheterization (84.9% *versus* 67.1%; p = 0.003). The median peak values of serum creatinine (1.84mg/dL *versus* 1.22mg/dL) and peak proBNP (9,461pg/mL *versus* 1,652pg/mL) were significantly higher in patients with *de novo* AF (p = 0.002 and p < 0.001, respectively).

**Table 4:** Complications, interventions performed, and laboratory markers according to the presence or absence of *de novo* atrial fibrillation

	*De novo* AF (n = 73)	No *de novo* AF (n = 237)	p value
Infectious complications			
Nosocomial infection	37 (50.7)	92 (38.8)	0.079
Sepsis	41 (56.2)	111 (46.8)	0.182
Septic shock	27 (37.0)	44 (18.6)	0.002
Interventions performed			
Catecholamine support	30 (41.1)	60 (25.3)	0.012
Non-invasive ventilation	13 (17.8)	30 (12.7)	0.332
Invasive mechanical ventilation	44 (60.3)	124 (52.3)	0.283
Days on invasive ventilation	2 (0 - 10)	1 (0 - 6)	0.082
Reintubation	6 (8.2)	7 (3.0)	0.086
Tracheotomy	8 (11.0)	15 (6.4)	0.205
Renal replacement	11 (15.1)	20 (8.4)	0.118
Central venous catheter	62 (84.9)	159 (67.1)	0.003
Laboratory markers			
Peak serum creatinine (mg/dL)	1.84 (1.09 - 3.65)	1.22 (0.89 - 2.41)	0.002
Nadir serum albumin (g/dL)	1.94 (1.55 - 2.42)	2.15 (1.65 - 2.64)	0.140
Peak C-reactive protein (mg/dL)	18.8 (9.81 - 28.8)	16.2 (5.8 - 28.8)	0.422
Peak proBNP (pg/mL)	9,461 (2,951 - 17,882)	1,652 (535 - 5,289)	< 0.001

AF - atrial fibrillation; proBNP - pro-B type natriuretic peptide. Values are expressed in n (%) or medians (interquartile ranges).

Patients with prior HF (n = 35, 11.3%) had significantly higher levels of proBNP than those without HF (median 9,017pg/mL *versus* 2,130pg/mL; p < 0.001). Considering the subgroup with *de novo* AF, the proBNP levels were not significantly different between patients with and those without HF (median 11,068pg/mL *versus* 7,875pg/mL; p = 0.222).

After the selection of the significant predictors in the univariate analysis and their application in the multivariable model (Table 5), the presence of stroke (OR = 10.09; 95%CI 1.54 - 66.27; p = 0.016) and elevated proBNP values (OR = 1.28; 95%CI 1.086 - 1.520; p = 0.004, for each 1,000pg/mL increment) were identified as independent predictors of *de novo* AF.

**Table 5:** Multivariate model for prediction of *de novo* atrial fibrillation

Multivariate model*	B	OR	95%CI for OR	p value
Age (per 1-year increment)	0.028	1.028	0.965 - 1.095	0.394
High blood pressure	0.936	2.550	0.482 - 13.487	0.271
Heart failure	0.997	2.711	0.447 - 16.438	0.278
Valve disease	19.818	4.04 x 108	0	0.999
Stroke	2.311	10.087	1.535 - 66.271	0.016
Thyroid dysfunction	2.407	11.105	0.784 - 157.2	0.075
APACHE II (per point increment)	0.140	1.150	0.990 - 1.336	0.067
SAPS II (per point increment)	0.062	1.064	0.987 - 1.146	0.104
Septic shock	1.584	0.940	0.872 - 1.013	0.162
Catecholamine support	0.528	1.696	0.247 - 11.624	0.591
Central venous catheter	0.239	1.269	0.157 - 10.292	0.823
Peak serum creatinine (per 1 mg/dL increment)	0.230	1.259	0.850 - 1.864	0.250
Peak proBNP (per 1,000 pg/mL increment)	0.250	1.284	1.086 - 1.520	0.004

B - coefficient B; OR - odds ratio; 95% CI - 95% confidence interval; APACHE II - Acute Physiology and Chronic Health Evaluation II; SAPS II - Simplified Acute Physiology Score; proBNP - pro-B type natriuretic peptide.

* Only variables with p < 0.05 were included in the multivariable analysis.

**Figure 2:**
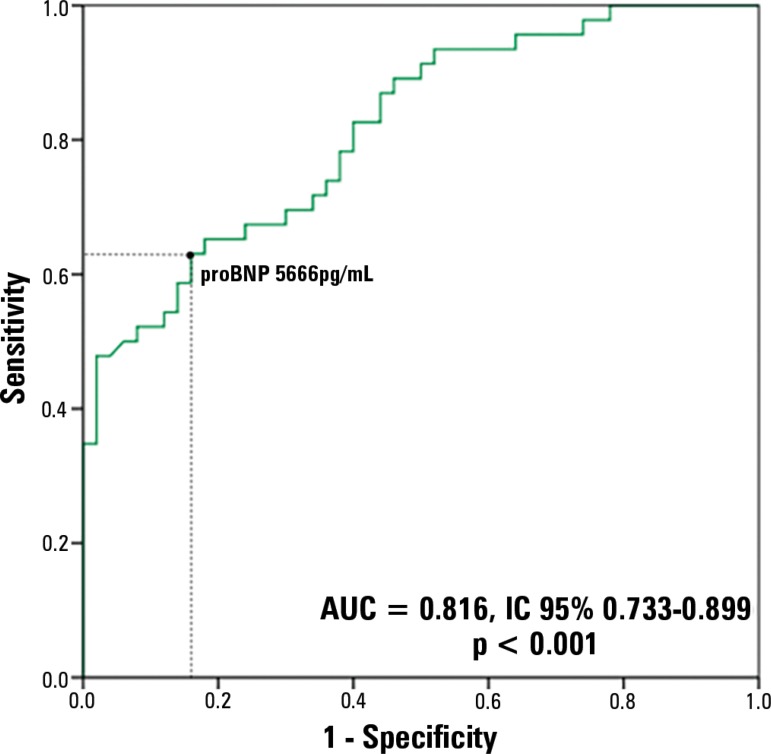
Receiver operating characteristic curve of peak pro-peptide natriuretic type B in the prediction of *de novo* atrial fibrillation. proBNP - pro-B type natriuretic peptide; AUC - area under the curve; 95%CI - 95% confidence interval.

The capacity of the proBNP peak to predict *de novo* AF during ICU stay was tested using the ROC curve; the area under curve (AUC) was 0.816 (95%CI 0.733 - 0.899; p < 0.001), demonstrating good performance (Figure 2). A proBNP value > 5,666pg/mL was identified as the optimal cutoff point for prediction of *de novo* AF, with a sensitivity of 65.2% and a specificity of 82% (Table 6).

**Table 6:** Performance of pro-B type natriuretic peptide > 5,666 pg/mL in the prediction of *de novo* atrial fibrillation

	Value	95% CI
Sensitivity (%)	65.2	62.2 - 68.2
Specificity (%)	82	79.5 - 84.3
Positive predictive value (%)	78.4	75.9 - 80.6
Negative predictive value (%)	70.2	68.3 - 72.1
Likelihood ratio for positive test	3.62	3.15 - 4.17
Likelihood ratio for negative test	0.42	0.39 - 0.46
Accuracy (%)	74.6	71.2 - 75.5

Patients with *de novo* AF had significantly longer stays in the hospital (14 [7 - 23] days *versus* 8 [4 - 19] days; p = 0.002) and ICU (8 [4 - 16] days *versus* 6 [3 - 12] days; p = 0.031).

There were no significant differences in in-hospital mortality between patients with and those without *de novo* AF (20.5 *versus* 15.6%; p = 0.370).

## DISCUSSION

### Predictors of *de novo* atrial fibrillation: the role of proBNP

In our population, the presence of previous stroke and an elevated proBNP value were independent predictors of *de novo* AF. The existence of previously documented paroxysmal AF is one of the possible explanations for the high prevalence of prior stroke in this subgroup with *de novo* AF. Such individuals presented sinus rhythm on admission, though they may have had previous paroxysmal AF that manifested *de novo* during hospitalization.

In turn, proBNP was found to be a marker with good performance in predicting *de novo* AF in the ICU. To the best of our knowledge, there are no previous studies demonstrating this role of proBNP in general ICUs. A recent study by Chokengarmwong et al.^(^^24^^)^ performed with 387 patients without AF revealed that proBNP at admission is a predictor of *de novo* AF in the first 3 days of hospitalization in a surgical and trauma ICU. In our study, proBNP > 5,666pg/mL showed good specificity and reasonable sensitivity in the prediction of *de novo* AF. However, the pathophysiological relationship between AF and proBNP still needs to be explained and may be attributed to atrial dilation, atrial fibrosis, or even decompensation of the underlying disease.^(^^25^^)^ However, it seems more likely that proBNP, like troponin, is a consequence rather than a cause of stress and/or injury. Regardless of the type of pathophysiological relationship between AF and proBNP, elevated values of the latter allow the identification of patients at risk for AF. In turn, the early identification of these patients allows establishing early strategies for the prevention of AF.

### High incidence of *de novo* atrial fibrillation in the general intensive care unit

The incidence of *de novo* AF observed in our medical non-cardiac surgical ICU was 23.5%, which is considered high in this type of ICU. Although several previous studies focused on cardiac and surgical populations,^(^^10^^-^^14^^,^^26^^)^ our data suggest that *de novo* AF is also a fairly frequent problem in the polyvalent ICU. Previous studies on the incidence of *de novo* AF in general ICUs have shown that the frequency of these events can reach 7 to 15%. However, some of these studies focused on the incidence of supraventricular tachyarrhythmias, regardless of the type of arrhythmia;^(^^4^^,^^17^^)^ in these studies, the incidence of AF may be lower.

The increased proportion of septic patients with nosocomial infection in the ICU during the period of our study may explain the high incidence of AF. In fact, inflammation is a common process in critically ill patients and may be a mechanism in the genesis of AF.^(^^27^^)^ In critically ill patients, in addition to the infectious pathology, respiratory and cardiac pathologies, invasive procedures, and the use of mechanical ventilation and catecholamine support may be triggers of AF.^(^^15^^)^

### Prognosis and prevention strategies

Previous studies have shown that AF is associated with higher in-hospital mortality in critically ill patients, especially in those with advanced age.^(^^28^^)^ Although there were no significant differences in in-hospital mortality between patients with and those without *de novo* AF in our cohort, the median days of hospital and ICU stay were significantly higher in the latter. To a certain extent, prolonged hospitalization in patients with AF may be associated with increased morbidity and higher health costs. Thus, the prevention of AF plays a central role in critically ill patients at increased risk (here identified by elevated proBNP). Several prophylactic AF strategies have been described,^(^^29^^,^^30^^)^ most of which are described in critically ill patients after thoracic surgery.

Our study has some limitations due to its retrospective nature and the heterogeneous group of patients. The small sample size and participation of a single hospital center also limit the capacity to infer the overall impact of AF predictors. Recording the type and dose of catecholamines administered was not part of the study protocol, and these data may have a relevant impact on the prediction of AF. Data regarding the position of the central venous catheter and the possible rapid volume expansion phases may play relevant roles in both the proBNP levels and the prediction of AF; however, these data were not evaluated in the present study. Although the diagnostic sensitivity of proBNP should be not be considered a strong effect, this limitation is compensated at least partly by the considerable specificity of proBNP in detecting *de novo* AF in this population. Only a small proportion of patients had available echocardiographic parameters; therefore, these data were excluded from the analysis. However, proBNP has the advantage of being an easily accessible marker in non-cardiac ICUs.

## CONCLUSIONS

History of previous stroke and elevated proBNP on admission were independent predictors of *de novo* atrial fibrillation in the polyvalent intensive care unit. ProBNP can be a useful and easily and quickly accessible tool to stratify the risk of atrial fibrillation. The high incidence of *de novo* atrial fibrillation in the polyvalent non-cardiac intensive care unit emphasizes the importance of timely recognition of this pathology.
